# Neuronal plasticity affects correlation between the size of dendritic spine and its postsynaptic density

**DOI:** 10.1038/s41598-018-38412-7

**Published:** 2019-02-08

**Authors:** Malgorzata Borczyk, Małgorzata Alicja Śliwińska, Anna Caly, Tytus Bernas, Kasia Radwanska

**Affiliations:** 1 Laboratory of Molecular Basis of Behavior, the Nencki Institute of Experimental Biology of Polish Academy of Sciences, ul. L. Pasteura 3, Warsaw, 02-093 Poland; 2 Laboratory of Imaging Tissue Structure and Function, the Nencki Institute of Experimental Biology of Polish Academy of Sciences, ul. L. Pasteura 3, Warsaw, 02-093 Poland

## Abstract

Structural plasticity of dendritic spines is thought to underlie memory formation. Size of a dendritic spine is considered proportional to the size of its postsynaptic density (PSD), number of glutamate receptors and synaptic strength. However, whether this correlation is true for all dendritic spine volumes, and remains stable during synaptic plasticity, is largely unknown. In this study, we take advantage of 3D electron microscopy and reconstruct dendritic spines and cores of PSDs from the *stratum radiatum* of the area CA1 of organotypic hippocampal slices. We observe that approximately 1/3 of dendritic spines, in a range of medium sizes, fail to reach significant correlation between dendritic spine volume and PSD surface area or PSD-core volume. During NMDA receptor-dependent chemical long-term potentiation (NMDAR-cLTP) dendritic spines and their PSD not only grow, but also PSD area and PSD-core volume to spine volume ratio is increased, and the correlation between the sizes of these two is tightened. Further analysis specified that only spines that contain smooth endoplasmic reticulum (SER) grow during cLTP, while PSD-cores grow irrespectively of the presence of SER in the spine. Dendritic spines with SER also show higher correlation of the volumetric parameters than spines without SER, and this correlation is further increased during cLTP only in the spines that contain SER. Overall, we found that correlation between PSD surface area and spine volume is not consistent across all spine volumes, is modified and tightened during synaptic plasticity and regulated by SER.

## Introduction

Dendritic spines are small protrusions on neurons that harbour synaptic receptors of excitatory connections. Since Ramon y Cajal described them more than 100 years ago, dendritic spines have been the focus of study of many neuroscientists due to their presumed role in the learning and memory processes^1–3^. At the tip of most of dendritic spines there is an electron-dense region that harbours glutamate receptors and other protein complexes, namely a postsynaptic density (PSD), which represents a postsynaptic part of a synapse^4^. PSD area was shown to correlate with dendritic spine volume and spine head volume^5–7^. Large PSDs and spines contain more AMPA and NMDA receptors, than small ones^8–10^. Hence, the greater the dendritic spine volume the larger PSD area and stronger the synapse^1,11^. Function and consequences as well as extent of this correlation are not fully understood.

Many studies analyzed the effect of synaptic stimulation on the size and shape of dendritic spines and PSDs (reviewed by Harris K.M. *et al*. in^12,13^ and Nimchinsky E *et al*. in^14^). The general view is that electrical and chemical LTP result in increased spine volume and PSD area^15–20^ and an increased frequency of non-macular synapses^16,17,20^. Synaptic plasticity-induced spine growth correlates with changes of AMPA and NMDA receptors-mediated currents^8,21–23^ and [Ca^2+^] transients^8^. However, to our knowledge, only two studies investigated if the activity-induced growth of PSD and dendritic is synchronous^24,25^. Meyer *et al*.^24^ and Bosch *et al*.^25^ used 3D electron microscopy (3D EM) to reconstruct dendritic spines with their PSDs, that underwent strengthening after glutamate uncaging, and showed that, as a result of a fast growth of a spine, there is a temporary shift in a ratio between the PSD surface area and spine volume. Due to limited sample size analysed so far, it is, however, unknown whether the correlation between a PSD and dendritic spine size is preserved during synaptic plasticity and whether the observed changes are universal across different dendritic spine sizes. Since it has been shown that small dendritic spines are very dynamic, whereas large mushroom spines tend to be more persistent^21,26,27^, the tight correlation between the spine volume and PSD area (and PSD volume) may not hold true for all spine categories. What is more, some dendritic spines contain smooth endoplasmic reticulum (SER), or its specialisation in form of stacks, called a spine apparatus. SER-containing spines are larger and have more potent synapses^23^. They are also preferentially enlarged during potentiation^28,29^, and necessary for long-term potentiation^28,30^. Still, little is known whether SER affects correlation between dendritic spine volume and PSD area.

In the current study we ask whether the correlation of PSD and dendritic spine size is an universal phenomenon across all spine sizes and if it is affected by synaptic plasticity and presence of SER. Toward this end we employed serial block-face scanning electron microscopy (SBEM)^31^ to obtain high-resolution, 3D measurements of dendritic spines with PSD-cores in the organotypic hippocampal slices after induction of chemical, NMDAR-dependent long-term potentiation (NMDAR-cLTP)^32^. The NMDAR-cLTP increased the volume of dendritic spines as well as volume and surface area of their PSD-cores. Moreover, the spines of the same volume tended to have larger PSD-cores, both in terms of area and volume, after stimulation, as compared to the control conditions. We also observe that the correlation between spine volume and PSD surface area and PSD-cores volume is not consistent across the whole spectrum of spine volumes, with medium-sized dendritic spines failing to show significant correlation of spine volume and PSD area or PSD-core volume. These correlations are, however, enhanced by cLTP. We further demonstrate that only spines that contain SER grow during cLTP, whereas PSDs growth irrespectively of the presence of SER. Overall, our research shows that correlation of PSD surface area and PSD-core volume with dendritic spine volume is not a strict rule and may be affected by the volume of dendritic spine, synaptic plasticity and SER.

## Results

### cLTP is associated with remodelling of dendritic spines and PSD-cores in OHCs

**Figure 1:**
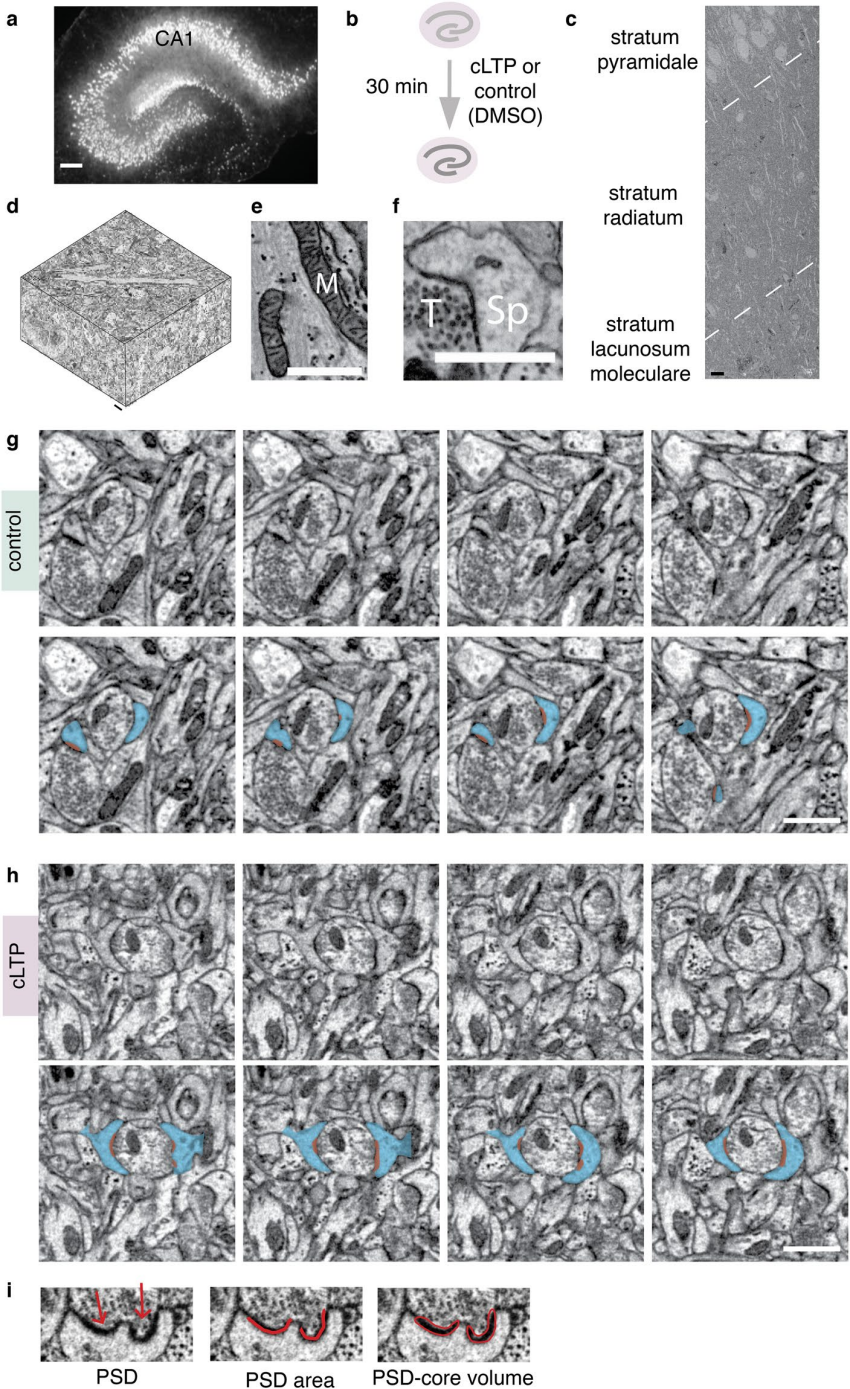
3D Serial Block-Face scanning electron microscopy of dendritic spines and core compartment of postsynaptic densities (PSD-core) in the organotypic hippocampal slices (OHCs). (**a**) Experimental design: OHC were treated with cLTP (n = 4) or DMSO (control, n = 4) for 30 min and then fixed. (**a**) OHC transfected with AAV1/2_CaMKII-mCherry to show its morphology. Scale bar, 200 μm. For 3D EM, only non-transfected OHC were used. **(c)** Low-magnification (5k) electron microphotograph of CA1 region of the organotypic slices showing conserved morphology. Dashed lines denote borders between strata. Scale bar, 10 μm. (**d**) 3D reconstruction of a full SBEM scan. Scale bar, 1 μm. (**e**,**f**) High magnification (25k) images of neuropil; M – mitochondrion, Sp – dendritic spine, T - axon terminal. Scale bars, 1 μm. (**g**,**h**) Consecutive images from SBEM scans with examples of dendritic spines from **(g)** control and (**h**) cLTP sample. Example dendritic spines are marked in blue and the PSDs are marked in red. Scale bars, 1 μm. (**i**) Example of a PSD segmentation (from a scan). Arrows indicate two parts of a perforated PSD. PSD-core volume was measured by outlining the electron-dense PSD core. PSD area was annotated with open traces along PSD opposing active zone (see methods for details).

To determine whether the correlation between the size of a synapse and dendritic spine volume is preserved during synaptic stimulation, we induced NMDAR-cLTP^32^ (Fig. 1a). We have used organotypic hippocampal slice cultures (OHCs), which preserve synaptic organisation of the hippocampal area CA1^33,34^ (Fig. 1b). OHCs were stimulated for 30 minutes with forskolin (50 μM), rolipram (100 nM), and picrotoxin (50 μM), or DMSO as a control, and then fixed. We chose this time point as forskolin-induced cLTP was shown to induce long-term growth of dendritic spines that sustained over 30 minutes^34,35^. It is, however, unclear whether enhanced synaptic transmission and spine enlargement is associated with the synchronised growth of PSDs in this model.

**Figure 2:**
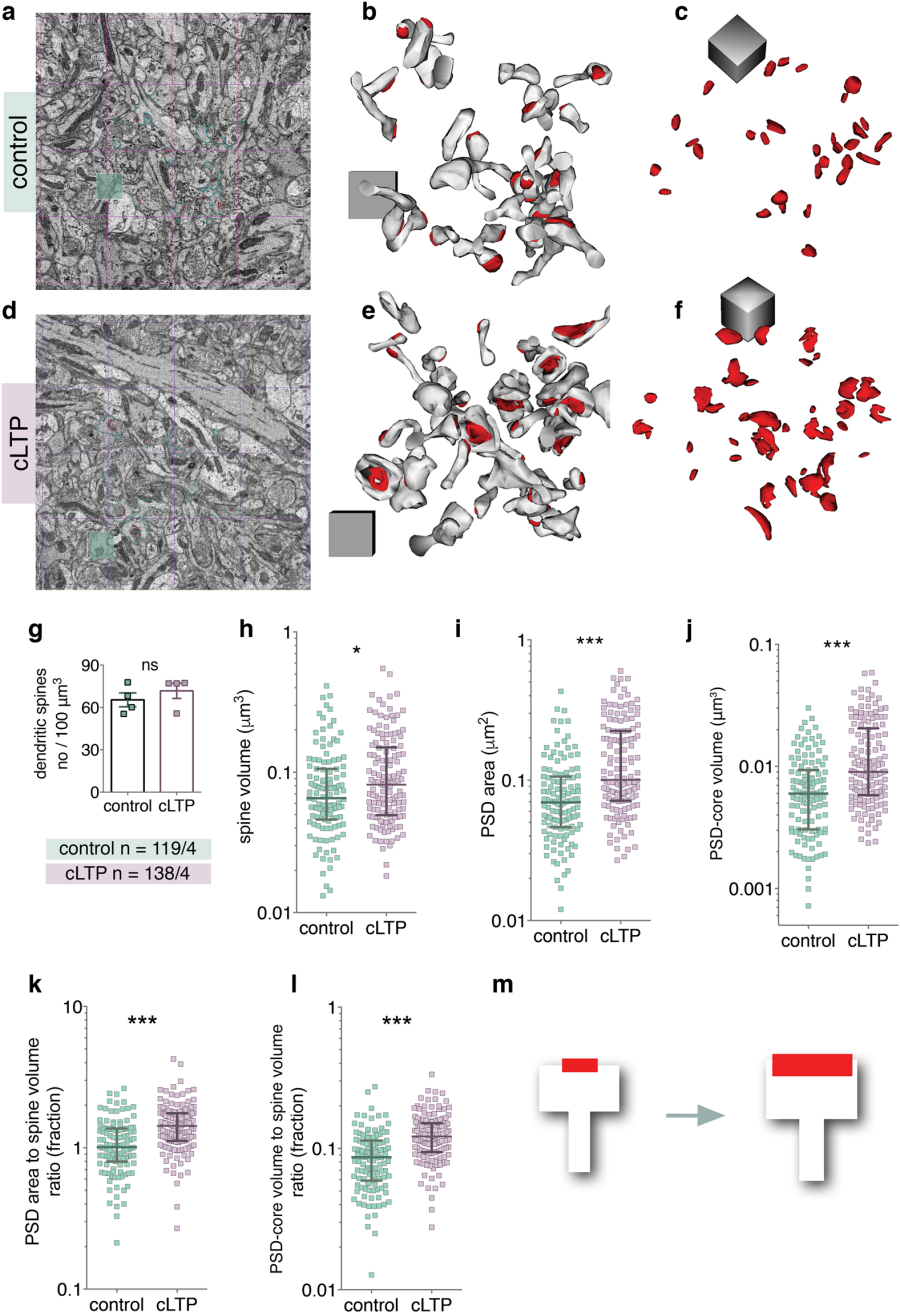
NMDAR-cLTP enlarges dendritic spines and their PSDs. (**a**,**d**) Fields of view of SBEM scan. (**b**,**e**) Example reconstructions of spines (white) with PDS-coress (red) from bricks of the control and cLTP-treated OHCs. Cubes are 1 × 1 × 1 μm. (**c**,**f**) PSD-core reconstructions from the same samples. (**g**) Spine density is not affected by cLTP (t-test, t(6) = 0.88, p = 0.41). (**h**) Spine volume is increased in the stimulated group (Mann-Whitney test, U = 6838, p = 0.02) as are the parameters of the PSD-core: (**i**) area (Mann-Whitney test, U = 4922, p < 0.0001) and (**j**) volume (Mann-Whitney test, U = 4969, p < 0.0001). (**k**) PSD area to dendritic spine volume ratio (Mann-Whitney test, U = 4708, p < 0.0001) and (**l**) PSD-core volume to dendritic spine volume ratio increases during cLTP (Mann-Whitney test, U = 4476, p < 0.0001). (**m**) cLTP in OHC is linked with slight increase of spine volume (white) and a large increase of PSD-core volume and area (red). *p < 0.05, ***p < 0.001. Mean ± SEM are shown for (**g**), median ± IQR for (**h**–**l**). Axes are log10.

To test this, we analysed dendritic spines and the core of PSDs^36,37^ (PSD-cores, but not pallium of PSDs, see also Materials and Methods for details), representing post-synaptic part of the synapse proximal to synaptic cleft, using the SBEM^31^. This technique is based on an installation of an ultramicrotome inside the microscope’s vacuum chamber and allows for 3D data collection with nano-scale resolution, from relatively big volumes of tissue. Strata of CA1 pyramidal neurons were distinguishable under low-magnification (Fig. 1c). 3D datasets were automatically collected from the medial part of the *stratum radiatum* of the CA1 area (Fig. 1d, see Supplementary Videos 1 and 2). High magnification images showed well-preserved membranes (Fig. 1e,f). For each organotypic slice, density of asymmetric PSDs on dendritic spines were analysed in four bricks of 150 μm^3^ using unbiased brick method^38^. Dendritic spines and their PSD-cores were reconstructed from a brick of 37 μm^3^ in each sample. Spine volume, PSD surface area opposed to the active zone and PSD-core volume, were manually segmented (Fig. 1g,h**)** and automatically measured by Reconstruct software^39^. PSD-core volume is used as a proxy of post-synaptic machinery which is proximal to synaptic cleft^36^. PSD surface area is an equivalent of the amount of available surface receptors^36,37,40,41^. Overall, 138 dendritic spines from stimulated slices (n = 4), and 119 from control slices (n = 4), were segmented by a trained annotator blind to the experimental conditions. Exemplary microphotographs and reconstructions of dendritic spines and PSD-cores from the bricks are shown for the control and cLTP OHCs in the Fig. 2a–f.

In consistence with the previous study^20^, density of dendritic spines was not affected by the chemical stimulation (Fig. 2g**)**. However, spine volume was significantly increased (Fig. 2h see Supplementary Table 1 for all analyses calculated per sample). PSDs had bigger surface area (Fig. 2i) and core volume (Fig. 2j). Next, we asked whether the increase in these parameters was proportional. Unexpectedly, the medians of the ratio between PSD area and spine volume, as well as PSD-core volume and spine volume, were higher in the spines that underwent cLTP, as compared to the controls (Fig. 2k,l). Thus, our analysis found that cLTP alters the ratio between PSD-core parameters (area and volume) and spine volume due to the growth of PSD-cores which exceeds the growth of dendritic spines (Fig. 2m).

### Correlation between dendritic spine volume, PSD surface area and PSD-core volume

**Figure 3:**
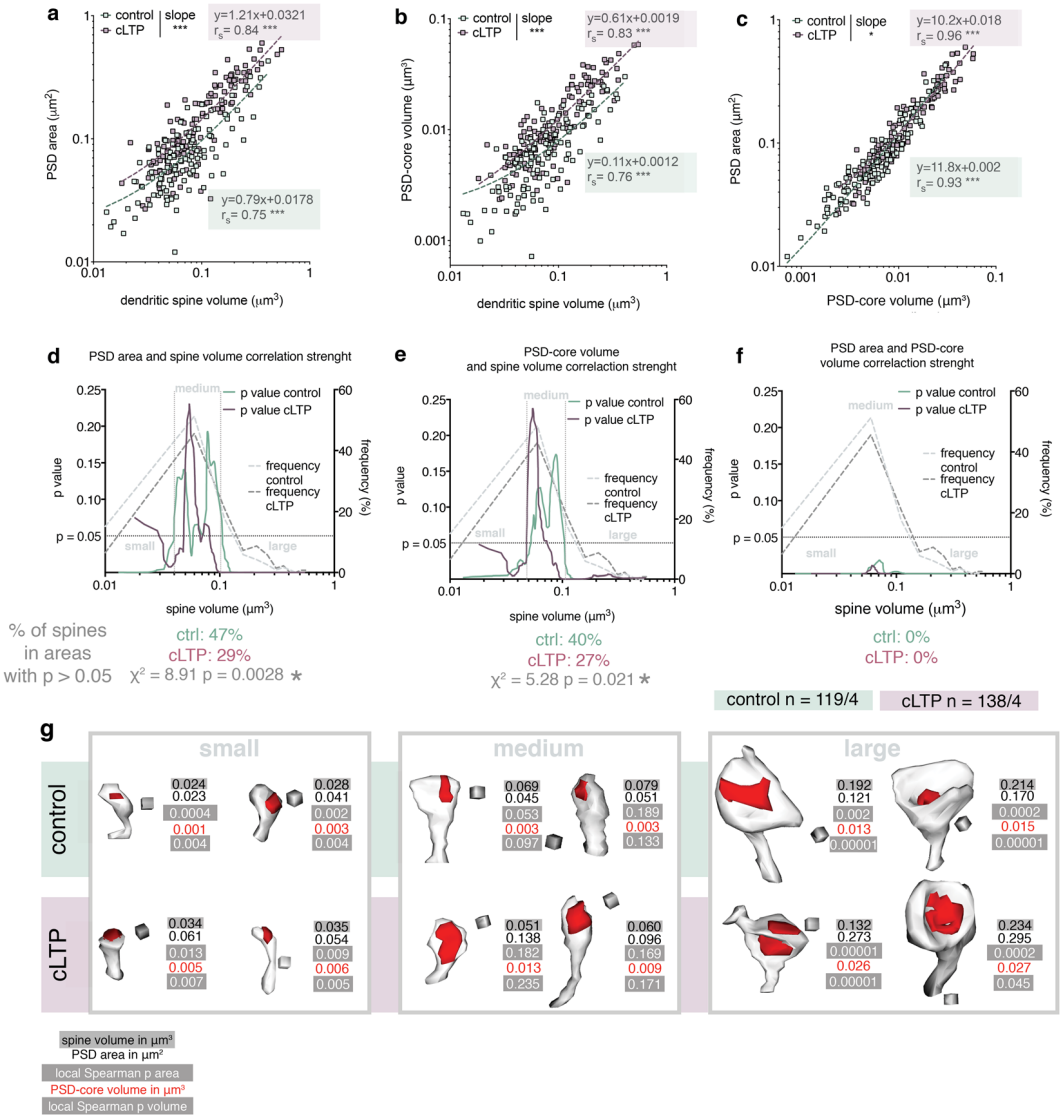
Dendritic spine volumes and PSD surface area and PSD-core volume are uncoupled in medium-size dendritic spines. (**a**) PSD area and spine volume tightly correlate (control: p < 0.0001, cLTP: p < 0.0001) and slopes of regression lines differ (ANCOVA, F_1,253_ = 55.40, p < 0.0001). (**b**) PSD-core volume and dendritic spine volume of spines from the control and cLTP-treated OHCs highly correlate with each other (control: p < 0.0001, cLTP: p < 0.001) and the slopes of the regression lines differ significantly (ANCOVA, F_1,253_ = 55.09, p < 0.0001). (**c**) PSD area and PSD-core volume show high correlation (control: p < 0.0001, cLTP: p < 0.0001) and slopes of regression lines differ between control and cLTP-exposed dendritic spines (ANCOVA, F_1,253_ = 6.44, p = 0.012). (**d**–**f**) Local values of Spearman correlation p calculated with sets of sliding windows around each dendritic spine (left y axis) for (**d**) PSD area versus spine volume; (**e**) PSD-core volume versus spine volume; (**f**) PSD area versus PSD-core volume; horizontal line depicts p = 0.05. Frequency (%) of spines in 0.05 μm^3^ volume bins is plotted on the right y axis. Presumed division lines between dendritic spine categories are indicated with vertical lines. Numbers of spines around which the correlation is not significant have been compared with Chi square test (below the graphs). (**g**) Example reconstructions of dendritic spines from the ranges discovered by the local correlation analysis. Parameters of each spine as well as local Spearmen p are given. Cubes are 0.0027 μm^3^. (**a–c**) Linear regression equations, Spearman correlation R and ANCOVA results are given for raw data, all graph axes are log10. (**d–f**) y axes are linear, x axes are log10.

Next, we analysed the correlation between the parameters of PSD-cores and dendritic spine volume. Overall, our data confirmed strong correlation between dendritic spine volume and PSD-core parameters (both surface area and volume), in the control and cLTP-treated population of dendritic spines (Fig. 3a,b). However, as expected from the ratio change (Fig. 2k,l), slopes of the regression lines for the control and cLTP-treated spines differed (Fig. 3a,b). We also analyzed the correlation between PSD surface area and PSD-core volume. As expected, they had coefficients of correlation near to 1. Moreover, slopes of the regression lines varied slightly between the control and stimulated population (Fig. 3c). Since the ratio between PSD-core volume and surface area may serve as an estimate of PSD-core thickness, the change in slopes of regression lines indicates thickening of the PSD-core during cLTP. All together these results show that in the general population the volume of a spine and PSD surface area strongly correlate, as previously described^6^.

Since earlier studies found that structural dynamics of dendritic spines depends on their volume^13,20,42^ we inquired whether the correlation of dendritic spine volumes and PSD-core parameters (surface area and volume), as well as coupling of these parameters during synaptic plasticity, is affected by spine size. To this end, we have developed a method to analyse local Spearman correlation strength across the whole spectrum of spine volumes. This was done by generating a set of windows (containing 14–24 spines) centred around each dendritic spine and calculating a mean p value of Spearman correlation locally within the window, using a resampling without replacement method. We have found that both for the PSD surface area and PSD-core volume, windows of spine sizes exist where correlation with dendritic spine volume fails to reach significance (Fig. 3d,e). For approximately 35% of dendritic spines the spine volume is a poor predictor of PSD area or PSD-core volume (Fig. 3d,e; p > 0.05 for correlation). In the control OHCs, these spine volumes fall mostly between 0.04–0.1 μm^3^ (Fig. 3d,e). In contrast, as expected, PSD area and PSD-core volume are very tightly correlated throughout the whole spine volume range, which serves as an internal control for the sliding window method use in this dataset (Fig. 3f).

Based on these results we propose that a morphologically distinct category of medium dendritic spines exists, where spine volume does not predict neither PSD surface area nor PSD-core volume. Illustrative of this is a phenomenon that spines of nearly the same volume, in the intermediate spine range, may have PSD-cores that differ 6-fold in their volume and 2.2-fold in their surface area (Supplementary Fig. 1). Such differences could not be observed among small and large spines.

Next, we evaluated whether the correlations of spine parameters was affected by the stimulation. Spearman correlation coefficient was higher for both PSD area and PSD-core volume vs spine volume in the group of the stimulated spines, as compared to the controls (Fig. 3a,b). Moreover, the range of spines were spine volume is a poor predictor of PSD-core size (both surface area and volume) is narrower in cLTP-treated group (0.048–0.073 μm^3^) (Fig. 3d,e). Indeed, when analysed with chi-square test the control spines have more points where the correlation function locally fails to reach significance, as compared to the spines after cLTP (Fig. 3d–f, below the graphs). Therefore, we conclude that upon stimulation coupling of the analysed spine parameters occurs.

### Role of Smooth Endoplasmic Reticulum (SER) presence in spine plasticity and correlation between dendritic spine volume, PSD area and PSD-core volume

**Figure 4:**
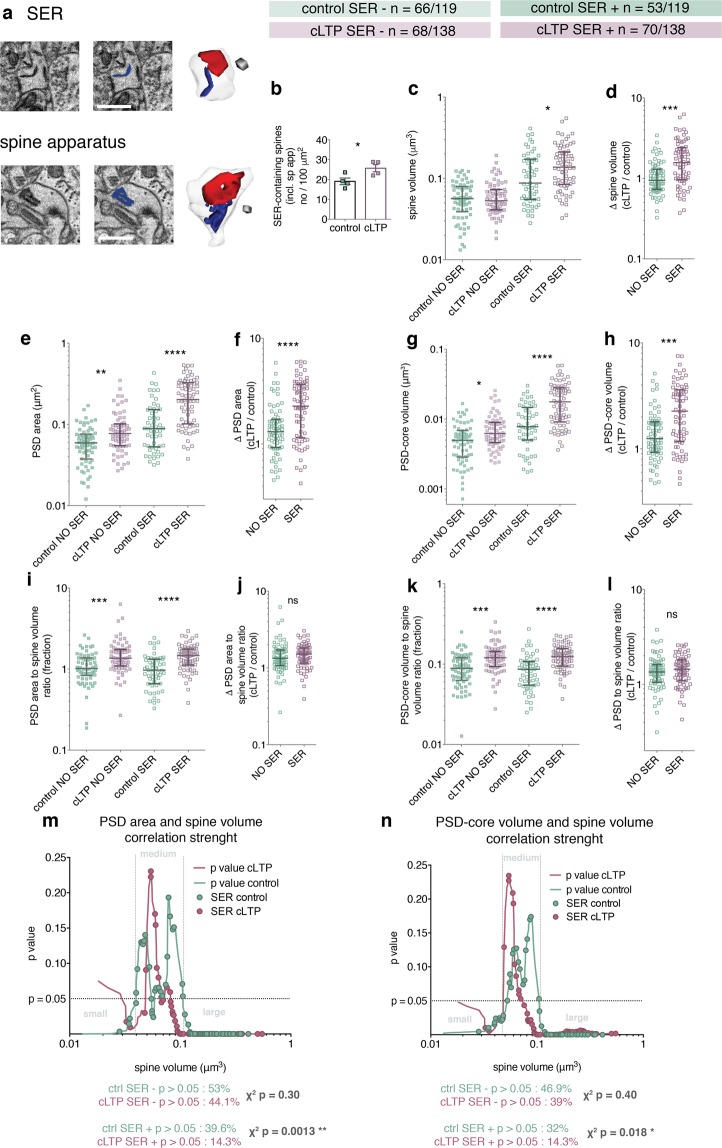
cLTP preferentially enlarges and enhances parameter correlation of dendritic spines with SER. (**a**) Example images of dendritic spines with a simple and complex (spine apparatus) SER tubules and their 3D reconstructions. Cubes are 0.0027 μm^3^, scale bars 1 μm. (**b**) Density of SER-containing spines is increased after 30 min of cLTP (t-test, t(6) = 2.68, p = 0.036). (**c**) Spine volume is increased after cLTP only in spines with SER (Kruskal-Wallis 78.35, p < 0.0001, Dunn’s post-hoc). (**d**) Relative spine volume change is higher in the group of dendritic spines with SER (Mann-Whitney test, U = 1412, p < 0.0001). (**e**) PSD area is larger after cLTP irrespectively of the SER content (Kruskal-Wallis 88.77, p < 0.0001, Dunn’s post-hoc). (**f**) PSD area increase is larger in SER-containing spines (Mann-Whitney test, U = 1423, p < 0.0001). (**g**) PSD-core volume increases in dendritic spines irrespectively of the SER content (Kruskal-Wallis 86.74, p < 0.0001, Dunn’s post-hoc). (**h**) PSD-core volume increase is more pronounced in the spines with SER (Mann-Whitney test, U = 1484, p = 0.0001). (**i**) PSD area to spine volume ratio increased in both SER-free and SER-containing dendritic spines (Kruskal-Wallis 38.72, p < 0.0001, Dunn’s post-hoc). (**j**) PSD area to spine volume ratio change does not differ between spine with and without SER (Mann-Whitney test, U = 2154, p = 0.34). (**k**) PSD-core volume to spine volume ratio is higher after cLTP for both categories of dendritic spines (Kruskal-Wallis 40.35, p < 0.0001, Dunn’s post-hoc). (**l**) Increase of PSD-core volume to spine volume ratio does not depend on SER-content (Mann-Whitney test, U = 2161, p = 0.35). (**m**,**n**) Local Spearman correlation p value for spines with SER (marked as dots on the curves). Number of dendritic spines with SER with non-significant correlation between spine parameters decreases after cLTP. The number of such spines without SER does not change (chi square tests below the graphs).

Dendritic spines with SER are thought to be preferentially recruited in various forms of synaptic plasticity, including LTP and LTD^23,28,43,44^. We therefore asked whether SER affects spine growth and correlation between dendritic spine volume and PSD-core size (both surface area and volume). We distinguished spines without or with SER (including both simple and complex tubules forming spine apparatuses) **(**Figs 4 and S2**)**. Density of spines with SER increased in cLTP-treated slices, as compared to the controls **(**Fig. 4b**)**. The increase of the spine volume after cLTP was significant only in the spines with SER (Fig. 4c). Thus, the relative change of spine volume after cLTP was higher for the spines with SER, as compared to the spine without SER (Fig. 4d). PSD area and PSD-core volume were larger after cLTP in both groups of dendritic spines (Fig. 4e,g), but the relative change of these parameters was again higher in the SER-containing protrusions (Fig. 4f,h). Both PSD area and PSD-core volume to spine volume ratio were increased in the stimulated groups (Fig. 4i,k**)** and that was independent of the presence of SER (Fig. 4j,l).

To check whether SER affected the correlation of PSD area and PSD-core volume with spine volume, we marked SER-containing spines on the local Spearman p curves (Fig. 4m,n**)** and found that after cLTP there is significantly lower frequency of the spines with SER that fall in the areas of insignificant correlation, as compared with the control. Such change was not significant for the spines without SER (Fig. 4m,n, chi-square test under the graphs**)**.

**Figure 5:**
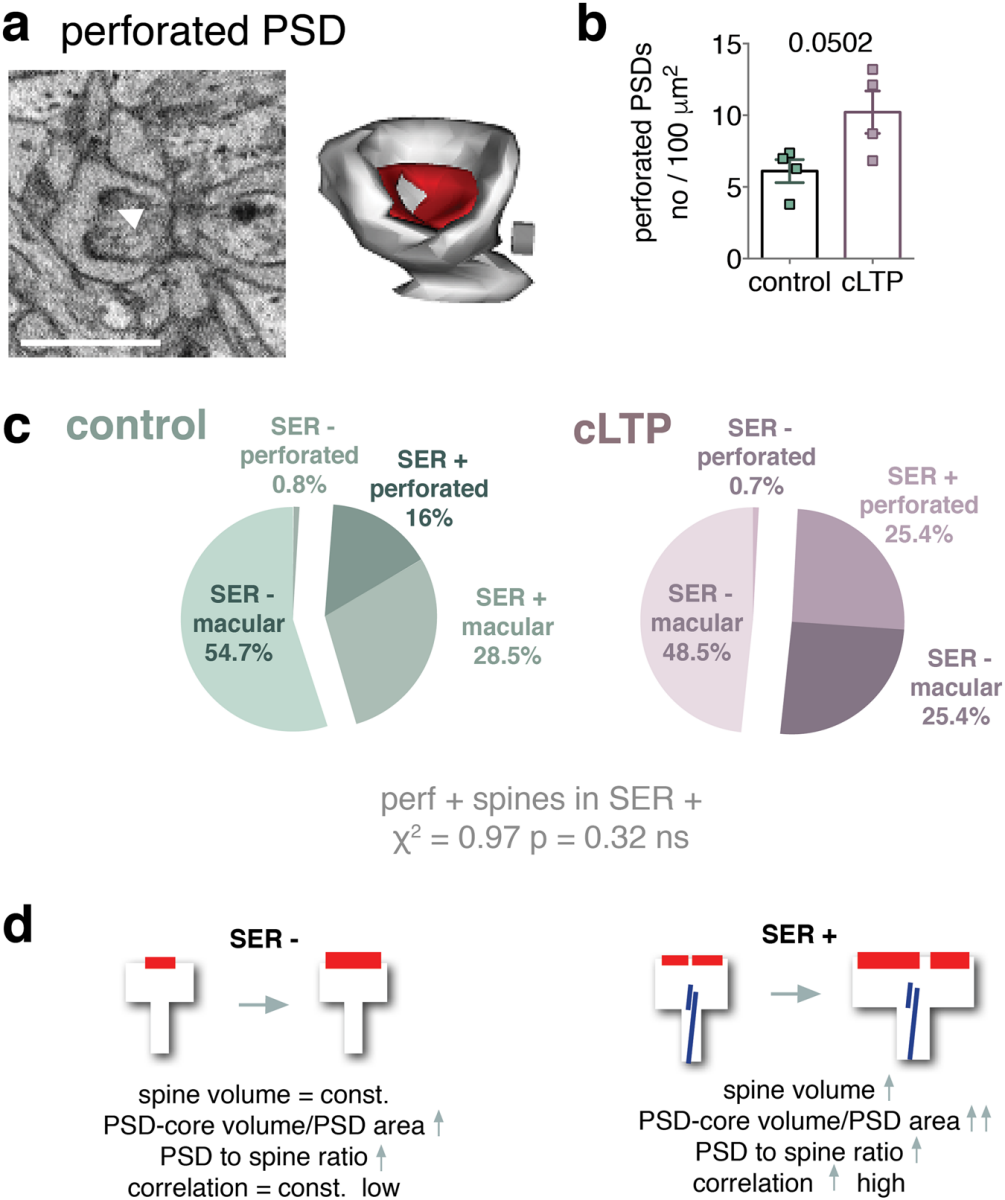
Spines with perforated PSD are a subset of dendritic spines with SER. (**a**) An example image of a perforated PSD (perforation indicated by white arrow) and 3D reconstruction of this dendritic spine. Cube: 0.0027 μm^3^. (**b**) Density of PSDs with perforation shows a tendency to increase during cLTP (t-test, t(6) = 2.44, p = 0.0502). (**c**) Almost all of dendritic spines with perforated synapses contain SER. Frequency of perforated synapses is not significantly increased during cLTP. (**d**) Schematic summary. Dendritic spines without SER show low correlation of spine parameters (spine volume vs PSD surface area and PSD-core volume) and increase only the size of PSD during cLTP. Spines with SER grow and increase the size of PSD during cLTP. They also show high correlation of the parameters, which is further tightened during cLTP.

Increased frequency of perforated and complex synapses has been linked with LTP^20^, however the function of the perforations is unknown. We therefore asked about the frequency and volume of dendritic spines with perforated PSDs. We found that there was a tendency for the perforated synapses to be more frequent after cLTP (Fig. 5b). We also found that perforated synapses almost exclusively belonged to SER-containing spines (with only 1 spine in each group that had a perforated PSD without SER occurrence) (Fig. 5c) but did not affect the probability of a spine or PSD growth (Fig. S3).

Taken together our findings revealed that during cLTP only spines with SER enlarge. Since density of the spines with SER is increased our data may also indicate, that the enlarging spines gain SER. At the same, PSDs grow both in spines with and without SER, but only spines with SER have perforated PSDs. Finally, PSD surface area, PSD-core volume and spine volume are more tightly coupled in the large spines, that contain SER, as compared to the spines without SER, and this coupling is further tightened during plasticity (Fig. 5d).

## Discussion

In this study, we employed 3D electron microscopy to reconstruct dendritic spines and their core PSDs in the hippocampal area CA1 of the organotypic slices after NMDAR-cLTP. We report that cLTP leads to larger spines only if they contain SER, while PSD-cores grow during stimulation irrespectively of the presence of SER in a spine. Thus, the growth of the PSD-core during potentiation, may be uncoupled from the growth of the spine leading to increased ratio of PSD-core parameters (surface area and volume) to spine volume. We also found that the correlation between PSD area and spine volume is not as fixed as previously regarded^5,6^. In the fraction of medium dendritic spines, where around 1/3 of the dendritic spines fall, the volume of spines does not predicts the parameters of PSD-core, neither its surface area nor volume. This correlation, however, increases after cLTP, especially in spines with SER. In conclusions, we propose that coupling of PSD-core dimensions to spine volume is an active process which is regulated by spine volume, its activity and SER.

### Growth of dendritic spines and their PSDs during cLTP

LTP induction results in structural remodelling of dendritic spines (reviewed in Bailey *et al*.^45^) and is associated with spine and synapse enlargement^16,24,34^. Here we confirm these observations showing that, after induction of a global cLTP, PSDs are enlarged in the stimulated spines and further clarify that spines grow only if they contain SER, a phenomenon that has been previously reported^28^. We also observed that the frequency of the dendritic spines with SER is increased after induction of cLTP. This observation may indicate that spines that grow gain SER. In spines that lack SER only PSDs are enlarged during cLTP. Our data is in concert with studies showing that spines that contain SER^23,44^, or spine apparatus (review by Segal *et al*.^29^), are more prone to plastic changes, possibly due to additional intracellular calcium release site^46^. Since larger PSDs contain more glutamate receptors^41^ our results also suggest that, in case of spines without SER, synaptic potentiation occurs without prominent growth of dendritic spines.

Perforated synapses have been given special attention as they are bigger and contain more AMPA and NMDA receptors than macular PSDs^47^. Our results indicate that nearly all perforated PSDs are associated with spines that contain SER. Since perforations do not predict spine or PSD growth we conclude that they may be a secondary feature of the large spines with SER which undergo profound remodelling.

### Correlation between spine volume, PSD surface area and PSD-core volume

Close structural relationship between a dendritic spine volume and area of its postsynaptic density is a broadly accepted phenomenon^5,6^. Here, we analysed PSD surface area and PSD-core volume and confirm that both of these parameters correlate with spine volume in the general population. However, we also observe that the correlation is not always true and depends on the spine volume. In basal conditions, moderate to strong correlations between PSD-core parameters and spine volume are present in small (approx. < 0.05 μm^3^) and large (approx. > 0.1 μm^3^) dendritic spines, while in the intermediate category of spines the volume of a spine rarely correlates with PSD area and PSD-core volume. The cut-off points for these subpopulations change after cLTP, in the way that the intermediate category is narrower and contains less spines. Moreover, SER-containing spines preferentially fall in the areas of significant correlation, suggesting that presence of SER and stimulation represent forces that drive dendritic spine volume and PSD surface area to become coupled. Our data suggest that during potentiation spines from the intermediate category acquire SER and their volume become tightly co-regulated with volume and area of PSD-core.

Thus far, coupling of PSD size to spine volume was perceived as a permanent spine feature^6^. Uncoupling of PSD and spine volumes, observed after glutamate uncaging, was suggested to be transient (<7 min), caused by slower growth of a PSD compared with a spine^24,25^. Here we show that PSD-core growth exceeds dendritic spine growth for at least 30 minutes after induction of chemical LTP. Therefore, the increase in PSD surface area to spine volumes ratio is not likely to be the result of slower remodelling of spines in relation to PSDs, but rather a long-lasting structural alteration and a way to regulate protein stoichiometry in dendritic spines. What is more, our data suggest that a change of PSD area to spine volume ratio, although similar in spines regardless of their SER content, may be less persistent in the spines that lack SER, as it results only from the change of PSD-core parameters without significant spine growth observed in dendritic spines with SER. To clarify this problem further studies are needed.

Precise reasons for dendritic spine volume and PSD-core volume correlation, and its coupling during synaptic plasticity, remain unknown^6,24,48^. Changes of the ratio between these two compartments most likely affect stoichiometry between proteins of the PSD and proteins of the dendritic spine, affecting properties of their complexes. An example of such link is that actin cytoskeleton is bound to cortactin which interacts with scaffolding proteins such Shank and Homer (via Shank)^49,50^. These, in turn, are connected with PSD-core scaffolding protein PSD-95, which provides slots for AMPA and NMDA receptors^51,52^. Computational models also predict that spine shape and size regulate Ca^2+^ dynamics^53^ and the threshold for synaptic plasticity^54^, while relationship between Ca^2+^ influx and spine size crucially determines long-term synaptic stability, synaptic strength and distribution of dendritic spine sizes^48^.

In conclusions, our study shows that relationship between dendritic spine volume and its PSD surface area and PSD-core volume is not as fixed as previously regarded. Our data show that an intermediate category of dendritic spines exists, where the area and volume of PSD-core are largely uncoupled from dendritic spine volume, and this is affected by synaptic activity and SER.

## Methods

The studies were carried out in accordance with the European Communities Council Directive of 24 November 1986 (86/609/EEC), Animal Protection Act of Poland and approved by the 1^st^ Local Ethics Committee in Warsaw, Poland (Permissions no 606_2014 and 438_2013). All efforts were made to minimize the number of animals used and their suffering.

### Organotypic Hippocampal Slice Cultures (OHCs)

Organotypic hippocampal slice cultures (OHCs) were established from the brains of the Wistar rats of both sexes on postnatal day 7 purchased from Mossakowski Research Center, Polish Academy of Sciences, Warsaw, Poland. OHC was prepared according to the interface method protocol by Gogolla *et al*.^55^. Briefly, the hippocampi were isolated from each hemisphere and cut into 300 µm-thick slices on a tissue chopper (McIlwain Tissue Chopper, Ted Pella). Only intact hippocampal slices were chosen for culture. Slices were cultured on UV sterilized, precut membrane pieces (#FHLC04700, Merck Millipore) on cell inserts (#PICM03050, Merck Millipore) in 6-well plates, for 14 days with medium exchange every third day. Slices were observed under a light microscope for the signs of necrosis and to see if the hippocampal regions were clearly distinguishable. For morphological visualization (only Fig. 1a) slices were transfected with adeno-associated virus, serotype ½ (AAV1/2) vector coding mCherry fluorescent protein under CaMKII promoter (obtained from Deisseroth’s Lab). The viral vector was added with a fresh medium (3 × 10^10^ viral particles/slice) to 7-day old slice culture and the medium was exchanged 72 h later. Slices were fixed in 4% PFA on 14 DIV, mounted with DAPI-containing mounting medium and imaged with fluorescent microscope (Leica AF7000).

### Chemical LTP in OHC

NMDAR-dependent, chemically-induced long-term potentiation of synaptic transmission (cLTP) was induced in OHC according to previously published protocol^32^ by adding forskolin 50 μM (F6886 Sigma), rolipram 100 nM (R6520 SIGMA), and picrotoxin 50 μM (P1675 SIGMA) to the medium on 14 DIV. As the chemicals were dissolved in DMSO (1,5 μl DMSO in 1 ml of culture medium), the same amount of the solvent was added to the medium of the control group. Slices were incubated with the solutions for 30 min and then fixed.

### SBEM sample preparation

Only slice cultures with healthy morphology were used for electron microscopy analysis. OHCs were fixed in 2% PFA (P6148 Sigma-Aldrich) with 2% glutaraldehyde (GA, EM grade, G5882 Sigma-Aldrich) in 0.1 M phosphate buffer pH 7.4 in ddH_2_O. Membrane pieces containing slices were taken off the insert with forceps and fixed ‘face down’ for 15 min in an ice-cold fixative. Afterwards, slices were rinsed with 0.1 phosphate buffer pH 7.4 (5 times for 3 min).

The SBEM staining was performed according to previously published protocol^56^. Slices were postfixed with solution of 2% osmium tetroxide (#75632 Sigma-Aldrich) and 1.5% potassium ferrocyanide (P3289 Sigma-Aldrich) in 0.1 M phosphate buffer pH 7.4 for 60 min on ice. Next, samples were rinsed 5 × 3 min with ddH_2_O and subsequently exposed to 1% aqueous thiocarbohydrazide TCH (#88535 Sigma) solution for 20 min. Samples were then washed 5 × 3 min with ddH_2_O and stained with osmium tetroxide (1% Osmium Tetroxide in ddH_2_O) for 30 min RT. Afterwards, slices were rinsed 5 × 3 min with ddH_2_O and incubated in 1% aqueous solution of uranyl acetate overnight in 4 °C. The next day, lead aspartate solution was prepared by dissolving lead nitrate (0.066 g) in 10 ml L-aspartic acid (0.998 g of L-aspartic acid (Sigma-Aldrich) in 250 ml of ddH2O). Slices were rinsed 5 × 3 min with ddH_2_O, incubated with lead aspartate for 30 min in 60 °C and then washed 5 × 3 min with degassed (autoclaved) ddH_2_O and dehydration was performed using graded dilutions of ethanol (ice-cold for better membrane preservation, 30%, 50%, 70%, 80%, 90%, and 2 × 100% ethanol (5 min each). Sample were infiltrated with a resin that was prepared by mixing: A (17 g), B (17 g) and D (0.51 g) components of Durcupan (#44610 Sigma-Aldrich) with 8 drops of DMP-30 (#45348 Sigma) accelerator^57^. Part of resin was then mixed 1:1 (v/v) with 100% ethanol and slices were incubated in 50% resin for 30 min in RT. The resin was then replaced with 100% Durcupan for 1 h in RT and then 100% Durcupan infiltration was performed o/n. The next day, samples were infiltrated with freshly prepared resin (as described above) for another 2 h in RT, then flat embedded between Aclar sheets (Ted Pella #10501-10). Samples were put in a laboratory oven for at least 48 h, 65–70 °C- for the resin to polymerize.

After resin hardening, Aclar layers were separated and the resin embedded samples were taken out. Squares, of approximately 1 mm × 1 mm, cut out with razor-blades, were attached to aluminum pins (Gatan metal rivets, Oxford instruments) with very little amount of cyanacrylate glue and then mounted to the ultramicrotome (Leica ultracut R) and trimmed. Samples were grounded with conductive silver paint (Ted Pella, 16062-15) to the pin and mounted into the 3 View chamber.

### 3 View imaging

Samples were imaged with SigmaVP (Zeiss) scanning electron microscope equipped with 3 View 2 chamber using a backscatter electron detector. Scans were taken in the middle part of *stratum radiatum* of the CA1 of the dorsal hippocampus, approximately 200 μm from the middle of the *stratum pyramidale*. From each sample 200 sections were collected (thickness 50–70 nm). Imaging settings: high vacuum with EHT 2.9–3.8 kV, aperture: 20 µm, pixel dwell time: 2 µs, pixel size: 7 nm (2048 × 2048 resolution).

### 3 View scan processing and image analysis

Scans were aligned using the ImageJ software (ImageJ- > Plugins-> Registration-> StackReg) and saved as.tiff image sequence. After alignment scans were then imported to the Reconstruct software^39^, available at http://synapses.clm.utexas.edu/tools/reconstruct/reconstruct.stm. True section thickness was determined using the cylindrical diameters method^58^ and was found to be consistent with the set thickness. Spine density was analysed with the unbiased brick method^38^ per tissue volume. For each sample all dendritic spines were counted in 4 bricks (first all synapses were found and then shaft and symmetrical synapses were excluded). Size of each brick was 10 μm × 10 μm × 1.5 μm. A structure was considered to be a dendritic spine when it was a definite protrusion from the dendrite, with electron dense material on a part of the membrane that opposed and axonal bouton with at least 3 vesicles within a 50-nm distance from the cellular membrane facing the spine. The electron-dense region is an approximate of the core of post-synaptic density (PSD-core) containing receptors as well as proximal scaffolding proteins of the postsynaptic part of a synapse (PSD-95/MAGUK filaments capped by guanylate kinase-associated proteins, GKAPs)^36,37,40,41^. PSD pallium, containing Homer and Shank proteins^37^, is rarely visible in a typical SBEM staining as ferrocyanide fixation leads to loss of pallium staining. The transition between PSD-core and pallium may be, however, dynamic as accumulation of CaMKII makes pallium more electron dense^37^.

Spine densities were averaged between bricks from each sample. Perforated PSD-cores and SER presence was annotated for each asymmetric PSD-core found. SER was defined as a dark tubule entering the neck of a given spine and Spine Apparatus was defined when at least two stacks of SER were visible adjacent to each other in the spine head.

For 3D reconstructions, from each sample a random brick was chosen and ¼ of its volume (5 μm × 5 μm × 1.5 μm) was subjected to 3D reconstructions. Brick thickness - 1.5 μm was sufficient to contain largest profile found in our dataset (0.93 μm in length) and the typical largest PSD found by others (about 0.7 μm in length^16,59^). First, PSD-cores found in this part were reconstructed and then their dendritic spines were outlined. PSD-core volume was measured by outlining dark, electron-dense area on each section containing PSD-core. PSD-core area was measured drawing a line along (if PSD-core was perpendicular to section) or outlining electron-dense area (if PSD-core was parallel to section). These two parameters of PSD-core were expected to be highly correlated, however, since they are subjected to different technical limitations (PSD-core volume measurement may be imprecise due to blurred borders of the PSD-core, while PSD-core area measurements due to software flat-contact calculations), we decided to measure both of them. To separate dendritic spine necks from the dendrites a cut-off plane was used approximating where the dendritic surface would be without the dendritic spine and terminating the spine on that surface. As we have based our reconstructions on the PSDs that we found in the brick, all non-synaptic protrusions were omitted in this analysis. For multi-synaptic spines we have summed the PSD areas and volume for these spines. Reconstruct software was used to extract spine parameters and generate 3D models. In total 257 dendritic spines with their PSDs were manually segmented by trained annotators blind to experimental conditions. The chief annotator (MS) was trained by Igor Kraev in the group of prof. Michael Stewart (OPEN University, UK) to identify and reconstruct dendritic spines and their PSDs.

### Statistical analysis of data

Statistical tests were performed in Graphpad Prism 6. Details of statistical tests are provided in figure legends. Dendritic spine volume, PSD-core volume and PSD area to spine volume ratio did not follow normal distributions and were compared with Mann-Whitney tests. For other parameters, unless specified, t-tests were performed. Correlations were analysed using Spearman correlation and difference between slopes or elevation between linear regression lines was calculated with ANCOVA. Differences between groups were considered significant if P < 0.05. For samples with normal distribution mean values and Standard Errors of the Mean (SEM) are shown; for samples which did not follow normal distribution medians and interquartile range (IQR) are shown.

### Local Spearman correlation analysis

Spine volumes (µm^3^) and corresponding values of their PSD surfaces (µm^2^) and PSD-core volumes (µm^3^) were converted to ranks using the former as the index vector for the latter parameters. Subsets of these values were selected with sets of sliding windows (comprising 14–24 dendritic spines) cantered at each value of the spine volume. The subsets were then resampled 2500 times, without replacement, to generate bootstrapped data sets, each containing 4 pairs of points less than the respective window size (10–20 elements). Rank correlation (Spearman) coefficients were calculated for the resampled (bootstrapped) data sets and averaged to produce local coefficient and p values corresponding to the spine volumes. Regions (ranges of spine volumes) where the p values were smaller than 0.05 were regarded as corresponding to significant correlation between the volumes and the surfaces.

## Supplementary information

SUPPLEMENTARY data

Suplementary video 1

Suplementary video 2

## Data Availability

The datasets generated and analysed in this study are available from the corresponding author on request.
