# Supplementary material for: Neuronal plasticity affects correlation between the size of dendritic spine and its postsynaptic density
Source: Sci Rep. 2019 Feb 8;9:1693. doi: 10.1038/s41598-018-38412-7 (PMC6368589; doi:10.1038/s41598-018-38412-7)
Supplement: Supplementary file 1 — SUPPLEMENTARY data [file 41598_2018_38412_MOESM1_ESM.pdf]

SUPPLEMENTARY MATERIAL

Neuronal plasticity affects correlation between the size of dendritic spine and its postsynaptic density

Malgorzata Borczyk[1], Małgorzata Alicja Śliwińska[1,2], Anna Caly[1], Tytus Bernas[2] and Kasia Radwanska*[,1]

[1] Laboratory of Molecular Basis of Behavior, the Nencki Institute of Experimental Biology of Polish Academy of Sciences, ul. L. Pasteura 3, Warsaw 02-093, Poland.

[2] Laboratory of Imaging Tissue Structure and Function, the Nencki Institute of Experimental Biology of Polish Academy of Sciences, ul. L. Pasteura 3, Warsaw 02-093, Poland.

* Corresponding author:

Dr. Kasia Radwanska, Nencki Institute of Experimental Biology, Polish Academy of Sciences, Warsaw, Poland, ul. L. Pasteura 3, 02-093 Warsaw, Poland; kradwans@nencki.gov.pl

Tel: (48 22) 58 92 252

Fax: (48 22) 822 53 42

Supplementary Video 1 & 2. Example scans from SBEM. Video 1 shows a scan from a control sliec. Video 2 shows a scan from a cLTP slice. Contrast was adjusted and Gaussian Blur of radius 1 px was applied.

Supplementary Table 1. Results of parameters shown in Figures 1-4 analysed per sample. Values are given as mean ± SEM of medians calculated from each sample (n=4/group)

	Control (mean ± SEM)	cLTP	t-test (df = 6)	
			p	t
spine volume (μm³)	0.067 ± 0.0078	0.080 ± 0.0086	0.27	1.21
PSD area (μm²)	0.06796 ± 0.006729	0.1093 ± 0.01519	0.0473*	2.49
PSD-core volume (μm³)	0.0056 ± 0.0008	0.0092 ± 0.0005	0.0093 **	2.27
PSD area to spine volume ratio	1.003 ± 0.05854	1.415 ± 0.06763	0.0037**	4.61
PSD-core volume to spine volume ratio (fraction)	0.085 ± 0.0071	0.12 ± 0.0059	0.0067 **	4.05
PSD-core volume to PSD area ratio	0.08090 ± 0.007045	0.08578 ± 0.002758	0.5427	0.64
spine volume SER - (μm³)	0.05880 ± 0.009938	0.05211 ± 0.004397	0.5609	0.615
spine volume SER + (μm³)	0.09651 ± 0.01023	0.1399 ± 0.0281	0.1966	1.45
PSD area SER - (μm²)	0.05321 ± 0.00761	0.07496 ± 0.00777	0.0925	2.00
PSD area SER + (μm²)	0.09116 ± 0.0153	0.2152 ± 0.0392	0.0259*	2.94
PSD-core volume SER - (μm³)	0.004380 ± 0.000879	0.006451 ± 0.000831	0.1380	1.71
PSD-core volume SER + (μm³)	0.008540 ± 0.00171	0.01713 ± 0.00355	0.0721	2.18
PSD area to spine volume ratio SER -	1.010 ± 0.03936	1.364 ± 0.1165	0.0282*	2.88
PSD area to spine volume ratio SER +	1.040 ± 0.09418	1.436 ± 0.04716	0.0094**	3.76
PSD -core volume to spine volume ratio SER -	0.08341 ± 0.00868	0.1172 ± 0.00809	0.0293*	2.85
PSD-core volume to spine volume ratio SER +	0.08536 ± 0.00899	0.1213 ± 0.00721	0.0206*	3.20

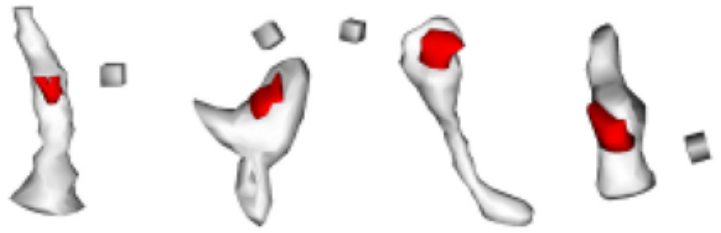

spine volume in µm³	0.076	0.077	0.073	0.077
PSD area in µm²	0.038	0.073	0.085	0.068
local Spearman p area	0.109	0.162	0.065	0.136
PSD-core volume in µm³	0.002	0.006	0.009	0.012
local Spearman p volume	0.083	0.101	0.054	0.091

Supplementary Figure 1. Range of PSD-core volumes and areas in spines of similar size. 3D reconstructions of dendritic spines (white) and their PSDs (red) with parameters of dendritic spines given below. There is a 6-fold difference in PSD-core volume and only 1.5% difference in spine volume between the first and last dendritic spine. Cubes are 0.0027 μm^3.

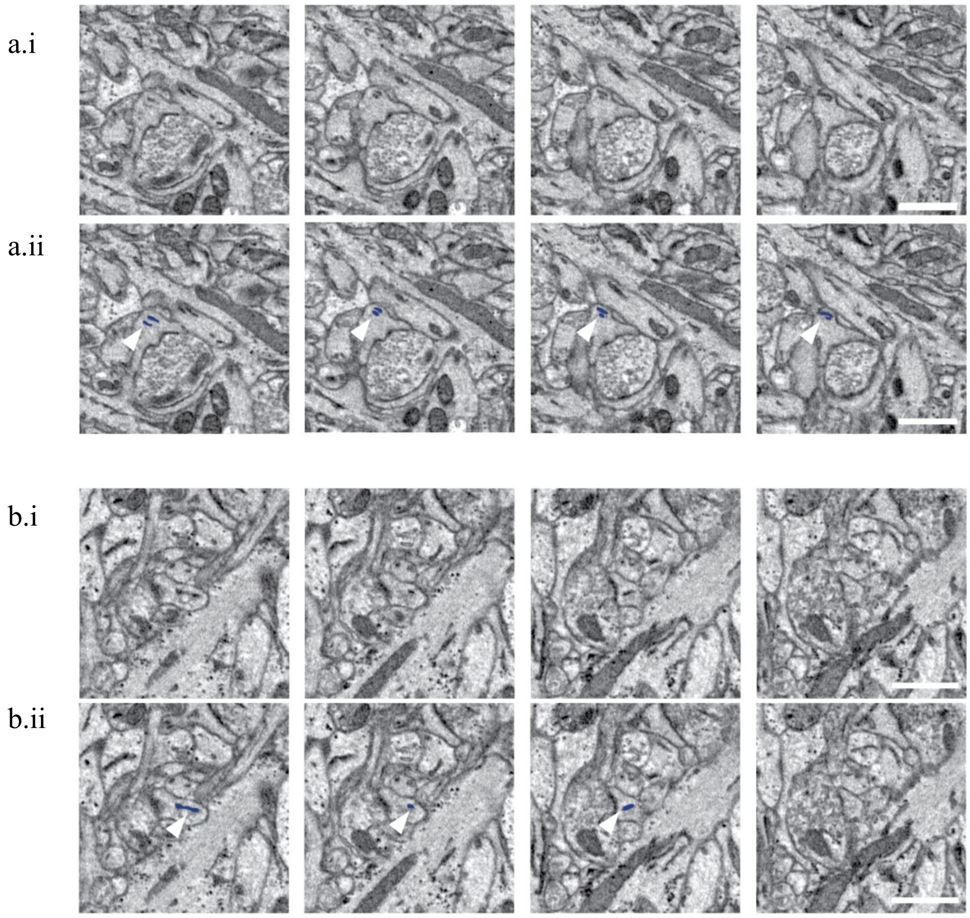

Supplementary Figure 2. Dendritic spines with SER and spine apparatus. (a) Consecutive EM images of a dendritic spine that contains spine apparatus, **(a.i)** images without annotations **(a.ii)** SER (in form of spine apparatus) is indicated in navy blue and white arrows. **(b)** Consecutive EM images of a dendritic spine that contains SER tubule, **(b.i)** images without annotations. **(B.ii)** SER is indicated in navy and with white arrow; scale bars 1 μm.

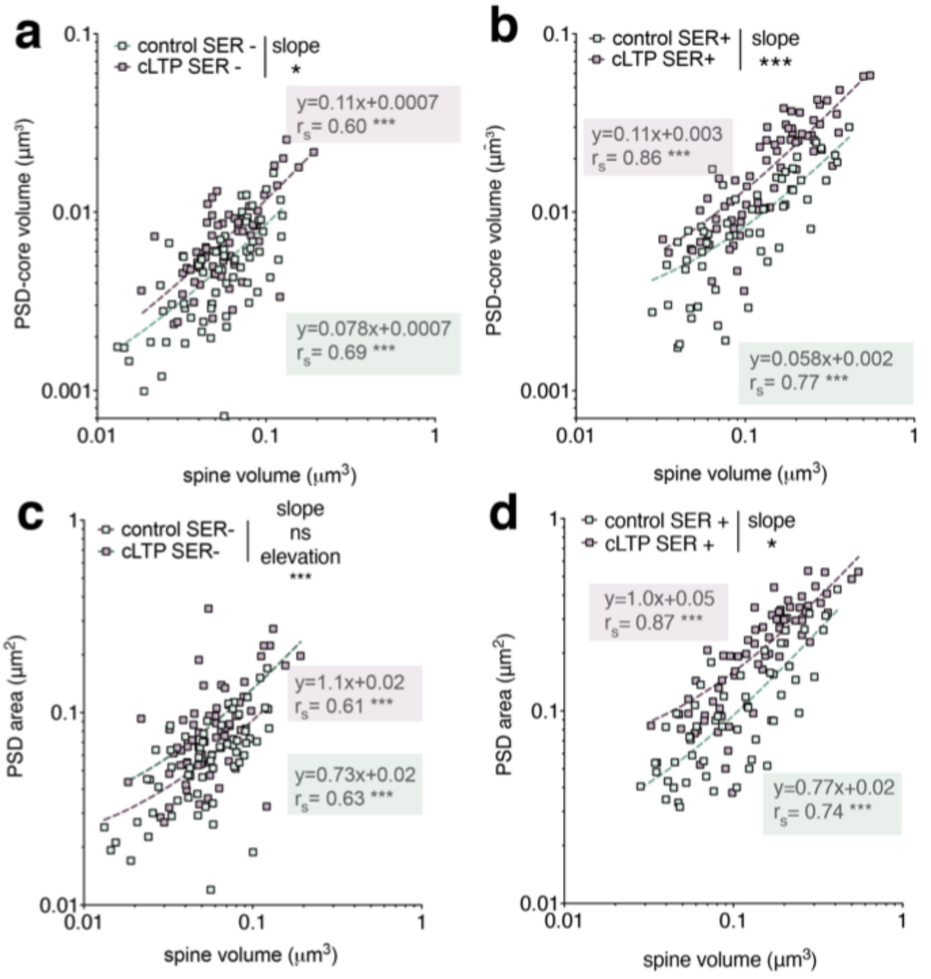

Supplementary Figure 3. Correlation between parameters of dendritic spines with and without SER. (a – d) In all four cases PSD-core volume and area and dendritic spine volume of spines from control and cLTP-treated OHCs correlate with each other, however Spearman r values are higher for spines that contain SER. Also, regression lines are different between the two groups in all cases shown. **(a)** Correlation between PSD area and spine volume for spines without SER (control: $p<0.0001$, cLTP: $p<0.0001$; slope difference: ANCOVA, $F_{1,130}=2.58$, $p=0.11$; elevation difference: ANCOVA, $F_{1,131}=17.56$, $p<0.0001$). **(b)** Correlation between PSD area and spine volume for spines with SER (control: $p<0.0001$, cLTP: $p<0.0001$; slope difference: ANCOVA, $F_{1,119}=6.08$, $p=0.015$). **(c)** Correlation between PSD-core volume and spine volume for spines without SER (control: $p<0.0001$, cLTP: $p<0.0001$; slope difference: ANCOVA, $F_{1,130}=4.062$, $p=0.049$). **(d)** Correlation between PSD-core volume and spine volume for spines with SER (control: $p<0.0001$, cLTP: $p<0.0001$; slope difference: ANCOVA, $F_{1,119}=21.42$, $p<0.0001$). All axes are log10.

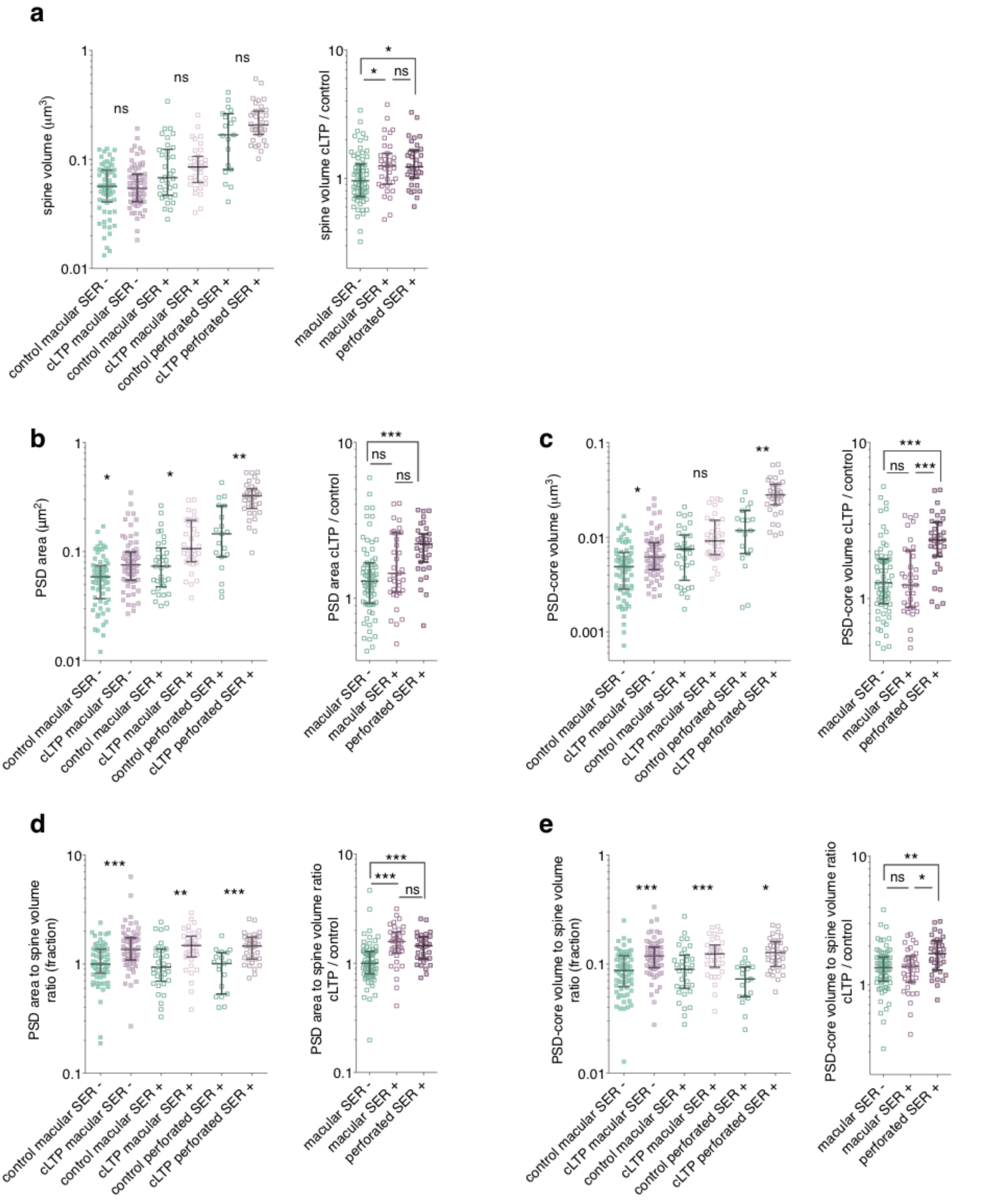

Supplementary Figure 4. Spine parameters dependence of perforation of the PSD and SER content. (a-e) Dendritic spines were divided into three categories – spines that do not contain SER, spines that contain SER and have a simple, macular, PSD and spines that both contain SER and a perforated PSD. Non-parametric Kruskal-Wallis test with Dunn's post-hoc was performed in all cases. **(a)** Spine volume ($H = 109$, $p < 0.0001$). Relative spine volume change is higher in the group of

dendritic spines with SER regardless of perforation ($H = 10.20$, $p=0.0061$). **(b)** PSD area is larger after cLTP irrespectively of the SER content and perforation ($H = 114.8$, $p < 0.0001$). Relative PSD area increase ($H = 19.29$, $p < 0.0001$). **(c)** PSD-core volume ($H = 110.2$, $p < 0.0001$). PSD-core volume increase is more pronounced in the spines with SER and perforation ($H = 25.55$, $p < 0.0001$). **(d)** PSD area to spine volume ratio increased in all three categories ($H = 39.94$, $p<0.0001$; relative increase: $H = 27.20$, $p<0.0001$). **(e)** PSD-core volume to spine volume ratio is higher after cLTP for all three categories of dendritic spines ($H = 43.88$ $p < 0.0001$). PSD-core to spine volume ratio increase does not depend on SER-content, but is more pronounced in dendritic spines with perforated PSDs ($H = 10.89$, $p = 0.0043$). Median +/- interquartile range are indicated, all axes are log10.
